# Another way? An investigation into an institution’s use of the Wayson stain in re-evaluating “no organisms seen” on Gram stain smears from positive blood cultures

**DOI:** 10.1128/spectrum.02573-24

**Published:** 2024-12-23

**Authors:** Christopher C. Attaway, Jessica Smith, Daniel D. Rhoads

**Affiliations:** 1 Department of Pathology and Laboratory Medicine, University of Vermont Medical Center 2092 , Burlington, Vermont, USA; 2 Pathology and Laboratory Medicine Institute, Cleveland Clinic 2569 , Cleveland, Ohio, USA; 3 Department of Pathology, Cleveland Clinic Lerner College of Medicine, Case Western Reserve University, Cleveland, Ohio, USA; 4 Infection Biology Program, Lerner Research Institute, Cleveland Clinic, Cleveland, Ohio, USA; Institute of Parasitology, Biology Centre, Ceske Budejovice, Czechia

**Keywords:** wayson stain, blood cultures, Gram-negative, bacteriology, clinical methods

## Abstract

Automated continuous monitoring blood culture instruments identify metabolism byproducts and flag blood culture bottles as “positive.” A Gram stain is used to visualize and characterize the microbial growth in the broth and initiate additional testing. When no organisms are seen (NOS) on Gram stain, in our laboratory, bottles are reevaluated with a Wayson stain, a rapid one-step stain that provides contrast between organisms and the background, especially in Gram-negative organisms. In this study, we assess the performance of the Wayson stain on reevaluation of Gram stain NOS blood culture bottles. The study period, August 2022 to July 2023, includes blood cultures that were flagged as positive by the automated blood culture instrument (BACTEC) with accompanying Gram and Wayson stain interpretations and organism identification. 133,463 blood cultures were performed during the study period, and 20,129 cultures were flagged as positive by BACTEC. 601 NOS Gram stain cultures were identified of which 76 had a positive Wayson. The Wayson stain procedure increased the sensitivity of direct organism visualization from 99.32% to 99.71% by identifying 55% (76 of 137) of NOS Gram stain blood culture bottles. Of blood cultures that were positive by BACTEC with NOS Gram and Wayson stains (475), 414 were finalized as no growth and 61 had an isolate (false negatives) of which 49% (30) were yeasts. The Wayson stain aided in detecting gram-negative genera such as *Capnocytophaga*, *Campylobacter*, and *Fusobacterium* species and other rarely identified species.

**IMPORTANCE:**

The Wayson stain, a rapid one-step stain that provides contrast between microorganisms and the background, was historically used for the presumptive diagnosis of *Yersinia pestis* from bubo specimen. In our laboratory, the Wayson stain has long been used to reevaluate blood culture Gram-stained smears from bottles that were flagged as positive by the automated continuous monitoring instrument but where no organisms are seen. In this study, we show that the Wayson stain provides an easily implemented and interpreted technique, other than a repeat Gram stain or acridine orange, to increase the sensitivity of direct organism visualization from blood culture bottles, particularly for Gram-negative organisms.

## INTRODUCTION

The Centers for Disease Control and Prevention estimate that there at least 1.7 million adults who develop sepsis each year in the United States (1). Timely identification of bacteremia associated with sepsis allows for improved clinical decision-making. Bacteremia is detected in the clinical laboratory by continuously monitoring blood cultures with automated systems that identify organism metabolism byproducts and flag blood culture bottles as “positive.” Subsequently, a Gram stain of the positive bottle is used to visualize and characterize the microbial growth in the broth, which initiates a cascade of additional laboratory testing with the goal of optimizing patient therapy and clinical management.

Automated continuous monitoring systems are subject to variables that dictate their success—blood volume, skin contamination, antimicrobials delivered before collection, delayed transportation to the laboratory, among others (2). Sometimes, despite being flagged as positive by the automated device, no organisms are seen (NOS) in the Gram stain. In some laboratories, these specimens are reevaluated with an additional Gram stain or a fluorescent acridine orange stain. If again no organisms are seen, these positive bottles are typically subcultured to solid media and incubated for several days, termed “blind subculture,” to determine if microbial colonies are detectable. In our laboratory, these Gram stain NOS bottles are reevaluated with a Wayson stain, which is a one-step stain comprised of a methylene blue, basic fuchsin, phenol, and ethanol, for the presence of organisms.

Historically, the Wayson stain, named after the early 20th-century physician researcher in the United States Public Health Service Newton Edward Wayson, was a modified methylene blue stain used for the presumptive diagnosis of *Yersinia pestis* from bubo specimens. The organisms on a Wayson stained smear are characterized by bipolar, safety pin-like staining which was highlighted by the stain among a purulent background (3). This morphology is appreciated on other aniline dyes such as Giemsa or Wright and among other members of the family *Enterobacteriaceae* (4, 5). Though the Gram stain utilizes aniline dyes for the primary and counter stain (crystal violet and safranin, respectively), the lack of retained crystal violet in the cell wall of Gram-negative bacteria and the weak staining of safranin precludes the evaluation of characteristic bipolar staining. Rather, organisms stained by Wayson appear deep blue, regardless of Gram staining, which provides contrast with the background and easier visualization, especially in Gram-negative organisms (6, 7). Of interest, the use of the Wayson stain in our laboratory precedes the authors by decades and the reason for its initial implementation over other methods remains elusive; however, in this study, we aim to assess the impact of using the Wayson stain as a secondary evaluation method of Gram stain NOS blood culture bottles.

## MATERIALS AND METHODS

This study was approved by the local Institutional Review Board (IRB). The laboratory information system (LIS) was used to identify adult (age >18 years at the time of collection) blood cultures performed from August 2022 through July 2023. A blood culture is defined as one collection, which is typically comprised of one aerobic (BD BACTEC, Plus Aerobic/F Culture Vials) and one anaerobic (BD BACTEC Lytic/10 Anaerobic/F Culture Vials) blood culture bottle. Specimens that were flagged as abnormal by BD BACTEC FX Blood Culture System (New Jersey, USA) along with Gram stain interpretation, Wayson stain (ENG Scientific, New Jersey, USA) interpretation, final organism identification, and methods used for identification were assessed. Final organism identification was accomplished by Vitek MS (bioMérieux, France), Bruker Biotyper MALDI-TOF MS (Billerica, Massachusetts, USA), or in-house partial 16S rRNA sequencing. Direct-from-blood culture MALDI-TOF MS and molecular identification panels are utilized in our laboratory for a rapid identification that is reported to the patient’s chart within a few hours of organism visualization from blood cultures; however, all blood cultures are plated on routine solid media for isolation, final identification, and antimicrobial susceptibility testing.

In our laboratory, the Wayson stain is proceduralized to be used as a secondary assessment of Gram stain NOS blood culture bottles, as opposed to a repeat Gram stain or acridine orange stain. The Wayson stain result (positive or negative) is not reported to the electronic medical record but is retained internally in the LIS for the isolate for documentation. The Gram stain smear, upon closer scrutiny with the knowledge of a positive Wayson stain, is reevaluated for Gram status and morphology before being reported to the patient’s chart, as this result is information on which ordering providers can interpret and act. Once an organism is visualized and classified (Gram-positive or Gram-negative), the isolate can proceed to rapid identification with MALDI-TOF MS or molecular multiplex panels, rather than reincubate the bottle and perform blind subcultures for a potential identification after 24 h of growth, thus improving turnaround time for organism identification.

Blood culture specimens in which no organisms are definitively seen by either Gram or Wayson stains are plated to routine solid media for incubation and manual inspection with the blood culture bottles returned to incubate in the automated continuous monitoring blood culture instrument.

## RESULTS

133,463 total blood cultures were performed during the 12-month inquiry period, and 20,129 were flagged as positive by BACTEC. 601 Gram stain NOS blood cultures were identified which received a follow-up Wayson stain: 475 were negative, 76 were positive, and 50 were excluded (quality control, proficiency testing, etc.). The positive isolates identified by Wayson staining and percent they represented of total isolates from the taxon recovered during the study period are displayed in Table 1. Of blood cultures that were positive by BACTEC with a subsequent negative Gram and Wayson stains (475), 414 were finalized with no growth after attempted subculture on routine solid media, and 61 resulted with an isolate identified that was not seen by either Gram or Wayson stains (false negatives, Table 1). Notably, yeast represented 49% (30) of NOS Gram and Wayson stained BACTEC positive blood culture isolates. The Wayson stain procedure increased the sensitivity of direct organism visualization from 99.32% to 99.71% by detecting 55% (76) of Gram stain NOS samples that contained an organism: 76 visualized by Wayson, 67 missed by Gram and Wayson stains, 137 isolates total.

**TABLE 1:** Percent of isolates visualized by Wayson stain of total positive blood cultures during the study period and isolates which were visualized neither by Gram stain nor Wayson stain

Isolate identification	% of isolate visualized by Wayson of total positive blood cultures (total number visualized by Wayson)	No. of isolates with “no organism seen” by Gram and Wayson stains (false negatives)
*Alistipes putredinis^a^*	100 (1)	
*Biophila wadsworthia*	100 (2)	
*Campylobacter jejuni*	100 (2)	
*Mycobacterium abscessus* species	100 (1)	
*Roseburia* species^*a*^	100 (1)	
*Sphingobium* species^*a*^	100 (1)	
*Capnocytophaga canimorsus^a^*	67 (4)	1
*Capnocytophaga sputigena*	67 (2)	1
*Aggregatibacter aphrophilus*	66 (4)	1
*Campylobacter fetus*	50 (1)	
*Delsulfovibrio desulfuricans*	50 (1)	
*Prevotella denticola*	50 (1)	
*Roseomonas mucosa*	50 (1)	
*Aerococcus sanguinicola*	33 (1)	
*Campylobacter ureolyticus*	33 (1)	
*Fusobacterium nucleatum*	28 (9)	1
*Eikenella corrodens*	20 (1)	1
*Gemella* species	17 (1)	
*Actinotignum schalii*	9 (1)	
*Fusobacterium* species	9 (1)	
*Haemophilus parainfluenzae*	8 (1)	
*Aerococcus urinae*	5 (1)	
*Staphylococcus saccharolyticus*	4 (1)	
*Haemophilus influenzae*	3 (2)	
*Candida albicans*	2 (4)	27
*Citrobacter freundii* complex	2 (1)	
*Streptococcus parasanguinis*	2 (1)	1
*Bacteroides fragilis* group	1 (1)	
*Candida glabrata*	1 (3)	3
*Candida parapsilosis* complex	1 (1)	
*Corynebacterium* species	1 (1)	1
*Enterobacter cloacae* complex	<1 (1)	
*Escherichia coli*	<1 (2)	1
*Klebsiella pneumoniae*	<1 (2)	
*Micrococcus luteus*	1 (1)	
*Staphylococcus aureus*	<1 (5)	6
*Staphylococcus capitis*	<1 (2)	
*Staphylococcus epidermidis*	<1 (3)	
*Staphylococcus hominis*	<1 (2)	2
*Streptococcus mitis-oralis* group	1 (3)	3
*Streptococcus pneumoniae*	<1 (1)	
*Actinomyces oris*		1
*Bacillus cereus* group		1
*Brevundimonas diminuta*		1
*Gordonia* species		1
*Peptoniphilus asaccharolyticus*		1
*Proteus vulgaris*		1
*Rothia* species		1
*Streptococcus anginosus* group		3
*Streptococcus sanguis*		2
Total	(76)	61

^*a*^ Denotes an isolate in which the final identification was achieved through 16s rRNA sequencing.

## DISCUSSION

**Fig 1:**
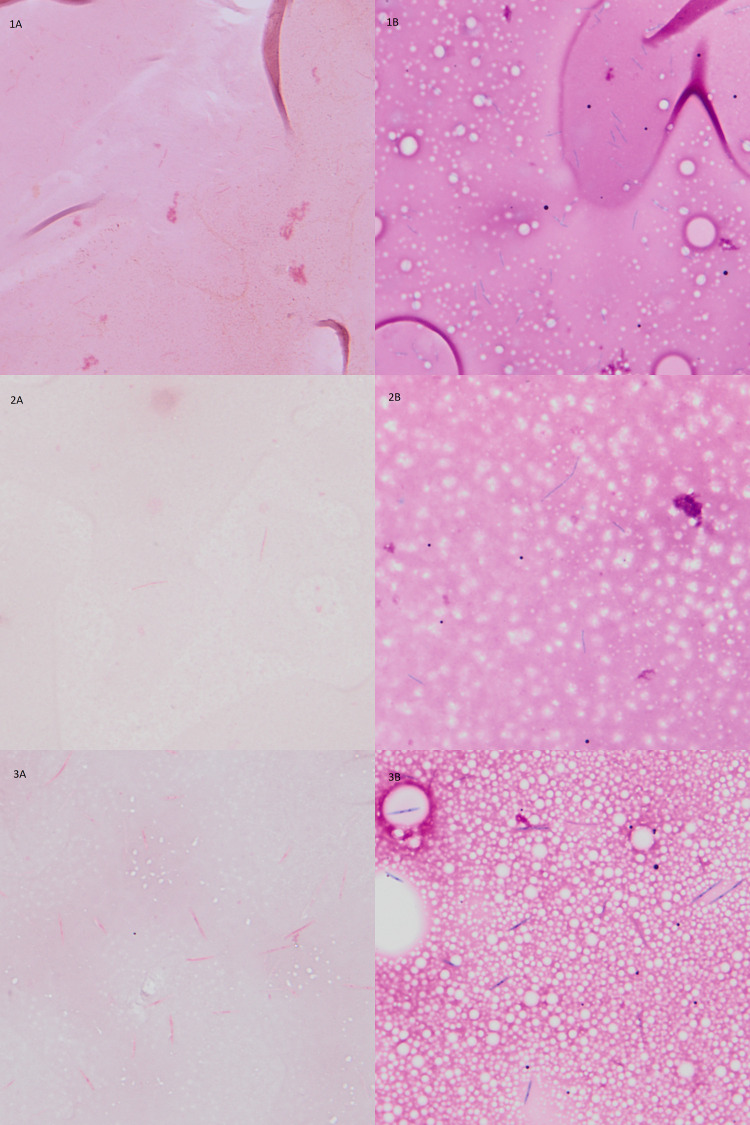
Photomicrographs comparing the Gram stains and Wayson stains of the following patient specimens: *Capnocytophaga* species Gram stain (1A) and Wayson stain (1B) and *Campylobacter* species Gram stain (2A) and Wayson stain (2B) initially not recognized on Gram stain with faint staining; *Fusobacterium nucleatum* Gram stain (3A) and Wayson stain (3B) initially interpreted as debris on Gram stain.

Visualizing an organism from the flagged blood culture bottle enables specimen progression to the next steps in identification and characterization in our lab, such as molecular identification by rapid multiplex blood pathogen panels, direct-from-blood culture MALDI-TOF MS identification, or rapid antimicrobial susceptibility testing from positive blood cultures (8–11). The Gram stain alone is the gold standard for visually identifying organisms from specimen smears. For Gram stain NOS blood cultures, the Wayson stain improved organism visualization in our study, and, to our knowledge, this is the first report of such for blood cultures. Particularly, the Wayson stain aided in visualizing major Gram-negative pathogenic organisms, especially anaerobes, fastidious, and weakly staining Gram-negative genera including *Capnocytophaga*, *Campylobacter*, *Fusobacterium*, and *Prevotella* species, as well as other rarely identified species (*Sphingobium* species, *Roseburia* species, etc.) (Table 1). Figure 1 shows side-by-side examples of challenging Gram stains in which organisms were not initially identified on Gram stain (Fig. 1: 1A *Capnocytophaga*; 2A *Campylobacter*) or were interpreted as artifact or debris (Fig. 1: 3A *Fusobacterium*) but are clearly visualized by the accompanying Wayson stain (Fig. 1: 3 A–C). These genera are united in their Gram-negative status and, usually long, thin morphology which may be difficult to identify on initial Gram staining. Furthermore, *Capnocytophaga* species grow optimally with increased CO2 concentrations (5%–10%) on enriched media and, in our experience, poorly for routine identification even under those conditions, as our final identification required 16S sequencing. *Capnocytophaga* species, most frequently *C. canimorsus*, are associated with severe septicemia under specific clinical context (asplenia, dog exposure) and have characteristic morphology (thin, long, fusiform rods); therefore, timely acknowledgment of the possibility of its infection through morphologic identification is critical (12). Fastidious *Campylobacter* species, with curved or gull-wing morphologies, prefer a microaerophilic atmosphere, a condition that blind subcultures of Gram stain NOS blood culture bottles would not routinely receive (13). Being able to appreciate the morphology at the time of the initial positive blood culture allows for appropriate growth conditions to be arranged.

The organisms which represented the greatest proportion visualized by Wayson (Gram-negative organisms) suggest that the Wayson stain itself allowed for greater sensitivity of blood cultures; however, other organisms, those which are normally easily identified by Gram stain alone (Gram-positive cocci), would suggest that a reassessment of the specimen, regardless of Wayson, allowed for the identification of these organisms. In many laboratories, a re-evaluation with a second Gram stain, rather than Wayson, would possibly yield similar results. The existence of a secondary review of the specimen itself may be a factor for the identification of isolates visualized by the Wayson stain though the Wayson stain offers additional contrast between organisms and the background and is accomplished in one step. In situations where the organism burden is low, reviewing more of the sample may be more important than the stain used. Our study echoes similar findings from laboratories that utilize other methods for reassessment of Gram stains, such as acridine orange, in the analysis of NOS blood cultures, with the Wayson stain having advantages when it comes to simplicity, with no requirement for fluorescent microscopy (14, 15). Institutions that utilized acridine orange described an increase in the stain’s ability to detect fungemia, a notable limitation of the Wayson stain in our study (14, 16). While some of the NOS Gram and Wayson stain identifications may be potential contaminants (coagulase negative *Staphylococcus* species), yeast comprised a large portion of NOS Gram and Wayson stains which presents opportunities for growth in our practice.

Beyond blood cultures, the Wayson stain has been used to increase the sensitivity of suspected *Haemophilus influenzae* type b meningitis in CSF specimens when compared to Gram stain by identifying small coccobacilli though vaccination has dramatically reduced the prevalence of this organism and the need for special stains when triaging specimens (7). The Wayson stain has also been used to identify other fastidious organism, such as *Helicobacter* species in gastric biopsies (17). However, one study hypothesized that Wayson staining could increase specificity for presumptive melioidosis detection but was unable to show a difference between Wayson and Gram staining in multiple specimen types (18). Of note, one study used a single, strong 1% carbol fuchsin stain (as opposed to 0.3% with traditional Gram stain), a component of the Wayson stain, compared against Gram stain to increase the sensitivity and specificity of *Campylobacter* in stool specimens (19).

Although the rate of false-positive flagging of blood culture bottles can be very low using advanced instrumentation, NOS findings still occur (20). Hyperleukocytosis, especially in cases of leukemia, are well-described causes (21, 22). Overfilling of culture vials may also contribute to positive blood cultures (23). However, some “false-positive” bottles, those in which no organisms are seen on initial Gram stain, may, indeed, contain organisms which are not appreciated, delaying additional results. In our laboratory, reassessment by the Wayson stain aided in triaging these initial NOS Gram stain blood cultures flagged positive by the automated system.

The Wayson stain, as per our procedure, is used when no organisms are seen by Gram stain on all blood smears; however, we acknowledge that an initial Gram stain NOS smear interpretation, in which organisms are indeed present, depends on the experience of the reader and quality of the stain. In our personal experience, the Wayson stain gives confidence in the interpretation of the Gram stain in challenging cases, an added benefit of performing an alternative stain for re-evaluation. This aspect is not measured in the current study, but, with variable levels of experience across the laboratory, present in all laboratories and among Gram stain readers, the Wayson stain offers an additional, different stain providing higher contrast between organisms and the background in triaging blood cultures to help prevent false negatives.
